# Evaluation of TP-E Interval and TP-E/QT Ratio in Panic Disorder

**DOI:** 10.3390/medicina56050215

**Published:** 2020-04-28

**Authors:** Abdulmecit Afsin, Ramazan Asoğlu, Mehmet Hamdi Orum, Elvan Cicekci

**Affiliations:** 1 Department of Cardiology, M.D, Kahta State Hospital, Adıyaman 02450, Turkey; 2 Department of Cardiology, M.D, Adıyaman Training and Research Hospital, Adıyaman 02450, Turkey; koroner63@yahoo.com; 3 Department of Psychiatry, M.D, Kahta State Hospital, Adıyaman 02450, Turkey; mhorum@hotmail.com (M.H.O.); mdrelvan@yahoo.com (E.C.)

**Keywords:** panic disorder, Tp-e interval, Tp-e/QT ratio

## Abstract

*Background and Objectives*: The autonomic nervous system (ANS) is involved in panic disorders. ANS dysfunction has been shown to be associated with ventricular arrhythmia and increased heterogeneity of ventricular repolarization. However, there remains limited evidence of the relationship between panic disorders and ventricular depolarization markers, including the Tp-e interval and Tp-e/QT ratio. This study aimed to evaluate ventricular repolarization parameters in patients with panic disorder. *Materials and Methods:* In total, 40 patients with panic disorder, diagnosed using the Diagnostic and Statistical Manual of Mental Disorders (DSM-5) criteria, were included in the study group. The control group comprised of 50 age- and sex-matched healthy individuals. A standard 12 lead electrocardiogram was recorded on all participants, and heart rate, QT interval, QRS duration, Tp-e interval, and Tp-e/QT ratio were measured. *Results:* QRS durations and QT intervals were similar in the study and control groups. Compared to the control group, QTd, Tp-e, and cTp-e intervals as well as Tp-e/QT and Tp-e/QTc ratios were significantly increased in patients with panic disorder (*p* < 0.05 for all). In the study group, the Severity Measure for Panic Disorder—Adult score had a significant positive correlation with the Tp-e interval (*r* = 0.369, *p* < 0001), cTp-e interval (*r* = 0.531, *p* < 0.001), Tp-e/QT ratio (*r* = 0.358, *p* = 0.001), and Tp-e/QTc ratio (*r* = 0.351, *p* = 0.001). *Conclusion:* These findings indicate that panic disorders are associated with increased ventricular repolarization heterogeneity, which may be attributed to ANS dysregulation.

## 1. Introduction

Patients frequently present with non-cardiac chest pain and palpitations in cardiology outpatient clinics. Non-cardiac chest pain and palpitations have a significant impact on an individual’s quality of life and are associated with mental health conditions [1]. Of patients with non-cardiac chest pain and palpitations, 72% have a coexisting mental illness. Moreover, patients with non-cardiac chest pain are particularly susceptible to experiencing panic attacks and developing panic disorders [2]. Previous studies have found that the lifetime prevalence of panic disorders varies between 1.7% and 2.7% [3].

The autonomic nervous system (ANS) regulates the heart rate and rhythm. It is well established that the ANS is involved in panic disorders. Symptoms such as chest pain, palpitations, sweating, breathing difficulties, feelings of suffocation, and hot flashes are common symptoms of panic attacks and indicate ANS dysfunction [4]. Moreover, it has been suggested that the ANS can cause changes in ventricular repolarization [5]. 

The QT interval and QT dispersion (QTd) are measured via a 12 channel electrocardiogram (ECG) to assess regional heterogeneity of ventricular repolarization. Studies have shown that ventricular repolarization heterogeneity increases the risk of cardiac arrhythmias [6]. Moreover, QTd is increased in patients with panic disorder [7]. Transmural dispersion of repolarization (TDR) occurs when ventricular repolarization does not occur simultaneously throughout the entire ventricle. Recently, T-wave measurements have been used to evaluate myocardial repolarization. The time between the T wave peak and T wave end (Tp-e) measured on an ECG can be used as an indicator for TDR [8]. Clinical studies have reported that the Tp-e interval and Tp-e/QT ratio are simple yet useful parameters for predicting increased ventricular arrhythmias and cardiovascular events [9]. However, there is limited evidence on the relationship between panic disorders and TDR markers such as the Tp-e interval and Tp-e/QT ratio. 

There are many clinical studies showing that the risk of death from cardiovascular diseases or cardiovascular causes is increased among individuals with panic disorder [10]. Therefore, this study aimed to evaluate ventricular repolarization parameters, including the Tp-e interval and Tp-e/QT ratio, measured on 12 lead superficial ECGs in patients with panic disorder.

## 2. Materials and Methods

Patients who presented to our outpatient cardiology clinic with panic disorder symptoms between 1 March 2019 and 30 September 2019 were eligible for participation. Panic disorder symptoms included palpitations, shortness of breath, sweating, anxiety, and fear. Patients who were referred to the psychiatry outpatient unit with the pre-diagnosis of panic disorder were included in the study group. A sample of subjects without cardiac symptoms served as the comparison group; volunteers were recruited from hospital staff. Healthy controls who had diagnosed cardiac or other organic disease, or who were using psychotropic or other medications were excluded. Demographic data as well as systolic and diastolic blood pressure values were collected from all participants. Current smoking status was recorded. Body mass index (BMI) was calculated by dividing weight in kilograms (kg) by height in meters squared (m^2^). Blood glucose, creatinine, thyroid-stimulating hormone, and electrolyte values were analyzed from blood samples collected at the initial clinic visit. Patients with acute coronary syndrome, stable coronary artery disease, hypertension, diabetes, kidney failure, left ventricular systolic dysfunction, heart valve disease, congenital heart disease, thyroid dysfunction, electrolyte disorder, and psychiatric disease were excluded from the study. Further excluded from the study were patients with rhythm disturbances or biphasic T and negative T waves on ECG, those using tricyclic antidepressants, antihistamines, antibiotics, and antipsychotics, and those that had previously used medication for a panic disorder.

In this study, panic disorder diagnosis was made according to the fifth version of the Diagnostic and Statistical Manual of Mental Disorders (DSM-5) (F41.0). Disease severity was determined using the Severity Measure for Panic Disorder—Adult (SMPD-A). The SMPD-A was developed by the American Psychiatric Association and is a self-assessment tool with 10 items, each scored on a five-point Likert-scale (0 = never, 4 = always). The SMPD-A asks participants to consider how the panic disorder has affected their personal lives in the past seven days. The resulting scores indicate the severity of the panic disorder. Total scores range from 0 to 40, with higher scores indicating more severe panic disorder. The Turkish version of the SMPD-A used herein has demonstrated good validity and reliability [11].

The 12 lead ECG recordings (50 mm/s, 10 mm/mV) were obtained in the supine position using a CardioFax S device (Nihon Kohden, Tokyo, Japan). Resting heart rate was measured using the ECG data. QT and Tp-e intervals and QRS duration were manually calculated by two cardiologists using the ECG data. Calipers and magnifying glasses were used to reduce measurement errors. The QRS duration was defined as from the time interval onset to the end of the QRS complex. The QT interval was calculated as the time from the start of the QRS complex to the end of the T wave. The longest QT interval from the 12 leads was defined as the maximum QT interval and the shortest QT interval was the minimum QT interval. QTd was calculated by subtracting the minimum QT interval from the maximum QT interval. The Tp-e interval was measured as the time from the T wave peak to the T wave end point on the precordial V5 lead [12]. The peak of the T wave was defined as a point of highest amplitude of T wave deflection. The end of the T wave was defined as the intersection of the tangent to the downslope of the T wave and the isoelectric line. If the V5 lead was not suitable for this measurement, the D2 lead was used. The measured values were corrected according to the heart rate using Bazett’s formula. Corrected QT interval (QTc), corrected QTd (cQTd), and corrected Tp-e interval (cTp-e) were obtained, and the ratios of Tp-e/QT and corrected Tp-e/QT (Tp-e/QTc) were calculated. The inter-observer and intra-observer variation coefficients for the Tp-e/QT ratio were 2.8% and 3.0%, respectively, and those for the Tp-e/QTc ratio were 2.9% and 3.1%, respectively.

Transthoracic echocardiographic evaluations were performed on all participants using a Vivid 5 Pro ECO device (General Electric, Horten, Norway). In the lateral decubitus position, the images of the parasternal long and short axis as well as four-chamber and two-chamber views from the apical window were obtained. The left ventricular ejection fraction was assessed using Simpson’s method [13].

Prior to enrolment, all participants received detailed information about the study and provided written informed consent. Research ethics approval was obtained from the Ethics Committee of Adıyaman University Medical Faculty (#17 September 2019, approved 17 February 2019).

### Statistical Analysis

All statistical analyses were performed using SPSS 22.0 statistical program (SPSS Inc., Chicago, IL, USA). Continuous variables were expressed as mean ± standard deviation and categorical variables were reported as numbers and percentages. The distribution of the data was assessed using the Kolmogorov–Smirnov test. Independent sample t-tests were used to compare continuous variables. Median, interquartile range (IQR), and Mann–Whitney U-tests were used for non-continuous parametric variables. Categorical variables were compared within the study group using chi-squared tests. Pearson correlation tests were used to evaluate the relationship between parametric continuous variables. *p*-values < 0.05 were considered statistically significant.

## 3. Results

In total, 40 patients with panic disorder (13 males; mean age, 34.7 ± 8.7 years) and 50 healthy controls (23 males; mean age, 34.5 ± 5.5 years) were included in the study. The demographic, laboratory, and echocardiographic data of all participants are summarized in Table 1. In patients with panic disorder, the average SMPD-A score was 21.2 ± 5.4. There were no statistical differences in demographic, laboratory, and echocardiographic variables between the two groups.

**Figure 1:**
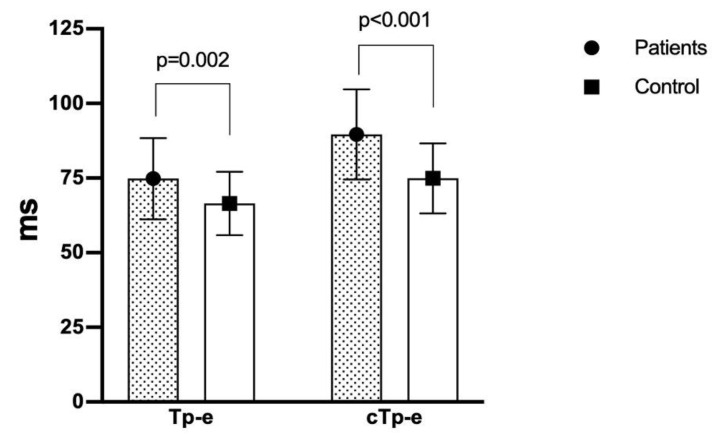
Comparison of Tp-e and cTp-e intervals in patients with panic disorder and healthy controls (74.8 ± 13.6 vs. 66.5 ± 10.6, *p* = 0.002; 89.6 ± 15.1 vs. 74.9 ± 11.7, *p* < 0.001, respectively). Tp-e: transmural dispersion of repolarization; cTp-e: corrected transmural dispersion of repolarization.

**Figure 2:**
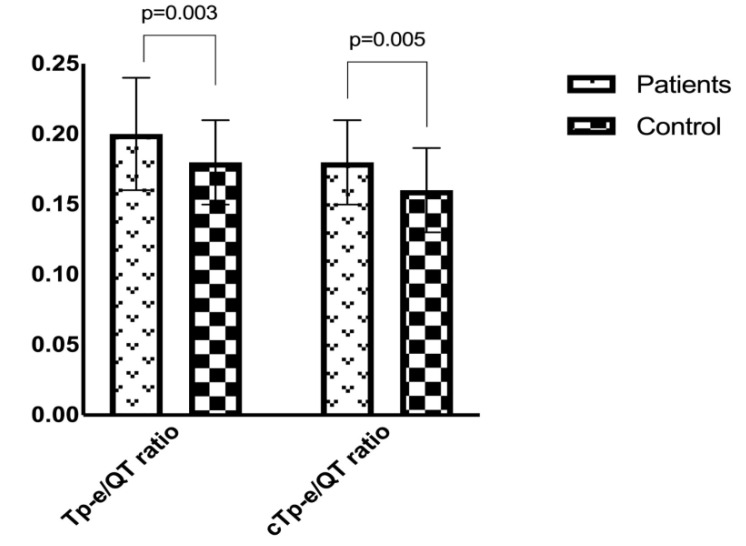
Comparison of Tp-e/QT and Tp-e/QTc rates between patients with panic disorder and healthy controls (0.20 ± 0.04 vs. 0.18 ± 0.03, *p* = 0.003; 0.18 ± 0.03 vs. 0.16 ± 0.03, *p* = 0.005, respectively). Tp-e: transmural dispersion of repolarization; cTp-e: corrected transmural dispersion of repolarization.

In the study group, the mean heart rate was 80.2 ± 13.6 bpm compared to 73.7 ± 11.2 bpm in the control group (*p* = 0.017). The QTd and cQTd intervals were significantly higher in the study group than in the control group (*p* < 0.05 for both). The study group had a significantly higher Tp-e interval (74.8 ± 13.6 vs. 66.5 ± 10.6; *p* = 0.002), cTp-e interval (89.6 ± 15.1 vs. 74.9 ± 11.7; *p* < 0.001) (Figure 1), Tp-e/QT ratio (0.20 ± 0.04 vs. 0.18 ± 0.03; *p* = 0.003), and Tp-e/QTc ratio (0.18 ± 0.03 vs. 0.16 ± 0.03; *p* = 0.005) (Figure 2) compared to the control group. A comparison of the electrocardiographic findings between the two groups is provided in Table 2.

In the study group, the SMPD-A score had a significant positive relationship with the Tp-e interval (*r* = 0.369, *p* < 0001), cTp-e interval (*r* = 0.531, *p* < 0.001), Tp-e/QT ratio (*r* = 0.358, *p* = 0.001), and Tp-e/QTc ratio (*r* = 0.351, *p* = 0.001). Correlations between the SMPD-A score and electrocardiographic parameters are presented in Table 3.

Logistic regression was performed to further investigate whether ECG variables have explanatory power over panic disorder. The results are presented in Table 4, and were in accordance with the findings of the t-tests which are shown in Table 2.

In Table 4 QRS duration, QTc, QTd, and Tp-e intervals as well as Tp-e/QT and Tp-e/QTc rates were statistically insignificant, while cQTd and cTp-e intervals were statistically significant. On average, the odds of a patient having panic disorder increased by 20% for each 1 ms increase in cTp-e interval, and the average odds of a patient having panic disorder increased by 5% for each 1 ms increase in cQTd interval. 

## 4. Discussion

This study evaluated electrocardiographic parameters among patients experiencing panic disorder symptoms compared to healthy controls. Participants in this study were not using any psychotropic drugs that could affect resting ECG results. A significant increase was observed in TDR markers, including the Tp-e interval, cTp-e interval, Tp-e/QT ratio, and Tp-e/QTc ratio, in the study group. To our knowledge, this is the first study to show variability in TDR among patients with panic disorder compared to healthy controls. These findings may indicate an increased risk of cardiac arrhythmia in patients with panic disorder.

Panic disorder has been associated with an increased risk of cardiovascular disease (CVD) and mortality [14]. Previous studies have shown a strong correlation between panic disorder and CVD, cardiomyopathies, arrhythmia, and decreased heart rate variability (HRV) [15]. Batelan et al. [16] investigated the effect of three anxiety disorders, panic disorder, social phobia–agoraphobia, and generalized anxiety disorder, on non-fatal CVD and found that many patients developed CVD during the 3 year follow-up period. The study also found a strong correlation between diffuse anxiety disorder and the onset of CVD. However, there remains limited evidence of any causal relationship between panic disorder and CVD [15].

The ANS is an important modulator of ventricular repolarization and plays a key role in the development and maintenance of malignant ventricular arrhythmias. However, the mechanism linking ANS dysfunction and ventricular arrhythmias is not yet fully understood [17]. Several clinical studies have shown an increased incidence of ANS disorders, including increased resting heart rate [18], reduced vagal tone, and increased sympathetic system efficacy [19], in patients with panic disorder compared to healthy controls. HRV is one method used to evaluate ANS function, which is controlled by parasympathetic and sympathetic cardiac nerves. HVR indicates the capacity of autonomic stimulation to be suppressed by the parasympathetic system [20]. A decrease in HRV is frequently used as an indicator of cardiac autonomic instability in panic disorder [21]. There are several models explaining the reduced HRV in panic disorder. According to the neurovisceral integration model [22], efferent nerve fibers from the prefrontal cortex moderate parasympathetic activity and vagal nerve inhibition of cardiac activity. This model defines a healthy functioning ANS with adaptive variability. This adaptive variability encompasses environmental, physiological, behavioural, cognitive, and emotional areas and plays a role in reducing anxiety that aggregates cardiovascular symptoms. Interventions that improve HRV may also help to ameliorate worry [23]. In their study of 41 patients with panic disorder, Gündüz et al. [24] detected decreased HRV parameters, suggesting reduced parasympathetic activity. Hovland et al. [25] reported that decreased HRV was associated with the severity of panic disorder symptoms.

QTd refers to the difference between the longest and shortest QT intervals measured on ECG. Clinical studies have shown that QTd is an independent factor in the QT interval among individuals with ANS dysfunction [26,27]. Increased QTd indicates heterogeneity in ventricular repolarization, which leads to ventricular instability [28]. Moreover, increased QTd has been associated with autonomic neuropathy and cardiovascular mortality [29]. However, there are few studies that evaluate the relationship between QTd and panic disorder. Atmaca et al. [7] found that the QTd was increased in patients with panic disorder compared to the control group. Although studies have suggested that increased QTd reflects an imbalance in the autonomic innervation of the heart and is associated with anxiety levels [30], others have shown that the QTd does not directly indicate ventricular repolarization heterogeneity [31,32]. In this study, the QT interval was similar in both groups, whereas the QTd interval was higher in patients with panic disorder.

Lubinski et al. [33] were first to report that patients with long QT syndrome had increased Tp-e intervals, which is an indication of TDR. It is well established that the Tp-e interval is more sensitive than QTd in assessing the heterogeneity of ventricular repolarization, as it reflects global dispersion, including apicobasal and interventricular repolarization dispersion. While the QT and Tp-e intervals are affected by BMI and heart rate, the Tp-e/QT ratio is not affected by these parameters and is more sensitive in predicting ventricular arrhythmias [34,35]. An increased Tp-e interval has been shown to be associated with mortality in Brugada syndrome, congenital or acquired long QT syndrome, hypertrophic cardiomyopathy, and ST-segment acute myocardial infarction [36]. Moreover, increased Tp-e interval and Tp-e/QT ratio have been associated with cardiovascular mortality [37]. A meta-analysis found that prolonged Tp-e intervals were associated with a 1.14-fold increase in risk of malignant ventricular arrhythmias or sudden cardiac death [38]. The authors suggested that the Tp-e interval might be a useful risk classification tool in various clinical populations as well as in the general public. In this study, the Tp-e interval and Tp-e/QT ratio were higher in the study group compared to the control group. This may be attributable to ANS dysfunction via increased sympathetic activity and/or decreased vagal tone during panic attacks. Moreover, changes in these parameters have been associated with increased ventricular repolarization heterogeneity and ventricular arrhythmias [39]. Therefore, these non-invasive ECG parameters may be used to assess the risk of adverse cardiac events in patients with panic disorder.

This study had several limitations. The incidence of arrhythmias and its relationship with arrhythmia markers were not assessed in this study. Due to the cross-sectional design, adverse cardiac events during clinical follow-up could not be evaluated. In addition, this study used 12 lead ECGs; however, the relationship between HRV and ventricular repolarization parameters may have been better assessed using 24 h rhythm Holter ECG recordings during panic attacks. 

## 5. Conclusions

In this study, ECG proxy markers for ventricular repolarization abnormalities, including QTd, Tp-e intervals, and Tp-e/QT ratios, were higher in patients with panic disorder compared to healthy controls. Secondary to panic attacks, electrophysiological properties of myocardial cells may change, resulting in altered repolarization characteristics in myocardial cells. Further large-scale, prospective studies are needed to evaluate the relationship between ventricular arrhythmias and increased Tp-e intervals and Tp-e/QT ratios.

## Figures and Tables

**Table 1:** Clinical characteristics and laboratory and echocardiographic findings of the groups.

	Panic Disorder Group (*n* = 40)	Control Group (*n* = 50)	*p*
**Baseline Demographic Parameters**			
Age (years)	34.7 ± 8.7	34.5 ± 5.5	0.188
Male sex, *n* (%)	13 (32.5)	20 (40%)	0.139
Married, *n* (%)	20 (50%)	20 (40%)	0.231
Smoking, *n* (%)	12 (30)	16 (32)	0.511
Body mass index (kg/m^2^)	26.8 ± 2.3	25.9 ± 2.6	0.099
Systolic BP (mmHg)	125 (118–130)	120 (120–125)	0.077
Diastolic BP (mmHg)	73.8 ± 7.9	70.9 ± 9.1	0.120
Severity Measure for Panic Disorder—Adult score	21.2 ± 5.4		
Laboratory Parameters			
Fasting glucose (mg/dL)	96 (86–99)	90 (86–95)	0.052
Creatinine (mg/dL)	0.84 ± 0.15	0.82 ± 0.13	0.484
Potassium (mmol/L)	4.2 ± 0.2	4.2 ± 0.3	0.484
Calcium (mg/dL)	9.57 ± 0.42	9.65 ± 0.47	0.434
Magnesium (mg/dL)	2.1 ± 0.1	2.0 ± 0.2	0.235
Thyroid-stimulating hormone (µU/mL)	2.33 ± 1.20	2.60 ± 1.12	0.055
**Echocardiography Parameters**			
LV ejection fraction (%)	55.3 ± 2.8	56.4 ± 3.1	0.080
LVEDD (mm)	43.7 ± 5.4	42.5 ± 3.6	0.190
LVESD (mm)	30.7 ± 4.2	30.9 ± 3.5	0.854
Left atrial diameter (cm)	3.7 ± 0.2	3.6 ± 0.3	0.197

BP: blood pressure; LV: left ventricular; LVEDD: LV end-diastolic dimension; LVESD: LV end-systolic dimension.

**Table 2:** Electrocardiographic findings of the study population.

	Panic Disorder Group (*n* = 40)	Control Group (*n* = 50)	*p*
Heart rate (beats/min)	80.2 ± 13.6	73.7 ± 11.2	0.017
QRS duration (ms)	86.7 ± 6.6	89.1 ± 6.3	0.094
QT interval (ms)	362.8 ± 24.9	361.9 ± 22.2	0.884
QTc interval (ms)	407.8 ± 21.2	405.1 ± 20.9	0.645
QTd interval (ms)	49 [40–60]	45 [40–60]	0.010
cQTd interval (ms)	62.4 ± 11.6	52.5 ± 17.3	0.004
Tp-e interval (ms)	74.8 ± 13.6	66.5 ± 10.6	0.002
cTp-e interval (ms)	89.6 ± 15.1	74.9 ± 11.7	<0.001
Tp-e/QT ratio	0.20 ± 0.04	0.18 ± 0.03	0.003
Tp-e/QTc ratio	0.18 ± 0.03	0.16 ± 0.03	0.005

QTc: corrected QT; QTd: QT dispersion; Tp-e: transmural dispersion of repolarization; cTp-e: corrected transmural dispersion of repolarization.

**Table 3:** Correlations between panic disorder scale and electrocardiography parameters.

	*r*	*p*
Heart rate (beats/min)	0.260	0.007
QRS duration	−0.151	0.082
QT interval	−0.030	0.391
QTc interval	−0.015	0.444
QTd interval (ms)	0.277	0.005
cQTd interval (ms)	0.294	0.003
Tp-e interval (ms)	0.369	<0.001
cTp-e interval (ms)	0.531	<0.001
Tp-e/QT ratio	0.358	0.001
Tp-e/QTc ratio	0.351	0.001

QTc: corrected QT; QTd: QT dispersion; Tp-e: transmural dispersion of repolarization; cTp-e: corrected transmural dispersion of repolarization.

**Table 4:** Logistic regression analysis results of investigation of the effect of ECG variables over panic disorder.

	Beta	*p*-Value	EXP (beta)	95% CI for EXP (beta)	
				Lower	Upper
BPM (beats/min)	0.059	0.147	1.060	0.980	1.148
QT	0.040	0.944	1.004	0.891	1.131
QTc	−0.109	0.166	0.897	0.769	1.046
QTd	0.056	0.071	1.057	0.995	1.123
cQTd	0.045	0.026	1.053	1.006	1.101
Tp-e interval	0.576	0.231	1.778	0.694	4.556
cTp-e interval	0.217	<0.001	1.242	1.103	1.400
Tp-e/QT ratio	−25.445	0.346	0.000	0.000	9.001
Tp-e/QTc ratio	−252.844	0.187	0.000	0.000	2.611
Constant	32.618	0.293	1.465		

CI: confidence interval; ECG: electrocardiography; QTc: corrected QT; QTd: QT dispersion; Tp-e: transmural dispersion of repolarization; cTp-e: corrected transmural dispersion of repolarization.
